# Self-assembled anchor layers/polysaccharide coatings on titanium surfaces: a study of functionalization and stability

**DOI:** 10.3762/bjnano.6.63

**Published:** 2015-03-02

**Authors:** Ognen Pop-Georgievski, Dana Kubies, Josef Zemek, Neda Neykova, Roman Demianchuk, Eliška Mázl Chánová, Miroslav Šlouf, Milan Houska, František Rypáček

**Affiliations:** 1 Institute of Macromolecular Chemistry, Academy of Sciences of the Czech Republic, Heyrovsky sq. 2, 16206 Prague 6, Czech Republic; 2 Institute of Physics, Academy of Sciences of the Czech Republic, Cukrovarnicka 10, 16253 Prague 6, Czech Republic; 3 Czech Technical University in Prague, Faculty of Nuclear Sciences and Physical Engineering, Trojanova 13, 12000 Prague 2, Czech Republic

**Keywords:** alginate, biomimetic surfaces, bisphosphonates, neridronate, poly(dopamine), spectroscopic ellipsometry, surface characterization, surface modification, titanium, XPS

## Abstract

Composite materials based on a titanium support and a thin, alginate hydrogel could be used in bone tissue engineering as a scaffold material that provides biologically active molecules. The main objective of this contribution is to characterize the activation and the functionalization of titanium surfaces by the covalent immobilization of anchoring layers of self-assembled bisphosphonate neridronate monolayers and polymer films of 3-aminopropyltriethoxysilane and biomimetic poly(dopamine). These were further used to bind a bio-functional alginate coating. The success of the titanium surface activation, anchoring layer formation and alginate immobilization, as well as the stability upon immersion under physiological-like conditions, are demonstrated by different surface sensitive techniques such as spectroscopic ellipsometry, infrared reflection–absorption spectroscopy and X-ray photoelectron spectroscopy. The changes in morphology and the established continuity of the layers are examined by scanning electron microscopy, surface profilometry and atomic force microscopy. The changes in hydrophilicity after each modification step are further examined by contact angle goniometry.

## Introduction

Titanium and titanium alloys are widely used in medicine and dentistry to replace and support hard tissues [1]. The absence of toxic alloying metals [1], extraordinary specific strength, appropriate Young’s modulus, outstanding biocompatibility and excellent corrosion resistance make commercially pure titanium a highly favored, biocompatible, metallic material [2]. The biocompatibility and corrosion resistance of titanium surfaces is closely related to the presence of a spontaneously formed 3–6 nm thick layer of titanium oxides, mostly in the form of titanium(IV) oxide (TiO_2_). The outermost surface of the oxide is covered with a 2.8–9.5 Å thick hydroxy group layer [3], which determines the reactivity of titanium surfaces [4] and sets their isoelectric point in the range of 3.5–6.2 [5–7].

Different surface modifications have been proposed to take the advantage of the titanium surface properties and to promote beneficial interactions at tissue–titanium implant interfaces. Established techniques use modifications of the titanium surface morphology and variations in the inorganic surface chemistry [8]. Procedures based on electrostatically driven adsorption [9–11], covalent coupling [12], electrochemical surface modifications [13], self-organized organic layers [14–15], etc. have been extensively studied for the immobilization of biologically active molecules [16] on titanium surfaces. Bio-related titanium surface modifications based on polysaccharides and synthetic polymers have been performed by physisorption and electrostatic interactions. In comparison with polylactide coatings, physisorbed alginate coatings are capable of exhibiting pronounced cell adhesion [17]. Chitosan/alginate, multilayered, 3D networks prepared by the layer-by-layer method enabled encapsulation of bone marrow stromal cells on the surface of dental or joint implants [18]. Polyelectrolyte (chitosan, poly(L-glutamic acid), and poly-L-lysine) coatings increased the surface ionic nature and wettability of the surface, yielding enhanced osteoblast differentiation [19].

The success of these modifications is highly dependent on the chemical state, reactivity and surface concentration of the hydroxy groups, as well as the presence of contaminants [12]. Therefore, one of the main objectives of this contribution is to perform and precisely characterize the activation of commercially pure titanium substrates for the realization of reactive titanium surfaces without contaminants. Such activated surfaces can be further functionalized by the covalent immobilization of self-assembled anchoring layers of different organic compounds, providing functional groups for further modification. Covalent bonding, which provides a stable fixation of immobilized compounds, is an alternative approach to coatings based on adsorption processes. The most common strategies for the formation of anchoring layers are thiol-based self-assembled monolayers (SAMs) [20] and silanes [21–22]. Despite the ease of preparation and high uniformity of the resulting layers, the thiol–SAMs provide an anchoring chemistry scheme limited only to noble metals. Furthermore, the established thiol bond is prone to oxidation and can be displaced from the surface [23–24]. Alkoxy- and chloro-silanes are widely used for the modification of different surface oxides. The mechanism of the layer formation includes replacement of a silane group by the transfer of a proton from the activated surface hydroxy group. This leads to the elimination of alcohol or hydrochloric acid, depending on whether alkoxysilane or chlorosilane, respectively, is used. In most cases the alkoxysilane treatment results in a 3D polymer network and extra precaution needs to be taken for the creation of a monolayer [22,25]. Surface treatments using 3-aminopropyltriethoxysilane (APTES) can result in several surface structures such as covalent attachment, self-assembly, multilayer formation by surface-initiated (SI) polymerization and particle adsorption [22]. The obstacles and limitations inherent to thiol–SAMs and silanes can be circumvented by the use of moieties bearing phosphonate [14,26–27] and bisphosphonate (BP) [28–29] groups. Upon hydrolysis, these form strong mono- and bi-dentate coordination bonds with metal surfaces [30]. Inspired by the composition of mussel adhesive proteins, Messersmith et al. [31] proposed the formation of poly(dopamine) (PDA) confluent films as a substrate-independent modification approach. The ability of PDA to adhere to solid surfaces stems from the reactivity of ortho-quinone/catechol moieties that form coordination bonds with surface metal oxides and covalent bonds with nucleophilic groups. In addition to this, the different PDA units can establish a wide range of non-covalent bonds through π stacking, hydrogen bonding, and van der Waals- and hydrophobic-interactions. PDA films have been used as the anchor layers of non-fouling polymer brushes [32–34], substrates for cell adhesion [35–36] and as platforms for controlled cell adhesion [37]. The presence of amine groups in PDA has been used for functionalization with moieties for photo-induced grafting reactions [38–39].

In this work, we study the immobilization of three compounds to the titanium surface: bisphosphonate neridronate, APTES and PDA. The neridronate covalent coupling leads to immobilization of the particular self-assembled molecules, whereas the immobilization of APTES or dopamine monomers results in the formation of partially or fully polymerized layers of APTES siloxane or PDA, respectively. The reactive amino end groups present in these anchor layers can be further utilized for the covalent bonding of a biofunctional coating of compounds bearing negatively charged functional groups. To this end, an anionic polysaccharide alginate extracted from the cell walls of brown algae (Phaeophyceae) was chosen as a model natural polymer, which satisfies the set prerequisites. This polysaccharide is biocompatible and degradable under normal physiological conditions [40] and has been used in various biomedical applications [41–42]. The presence of carboxyl groups in the structure of β-D-mannuronate and α-L-guluronate monomer units can be utilized for the immobilization of the polysaccharide chains to the anchor layer amine groups through the creation of amide bonds.

The success of the performed modifications and their short-term stability in a phosphate buffer at 37 °C was probed by different surface sensitive techniques such as X-ray photoelectron spectroscopy (XPS), spectroscopic ellipsometry (SE) and infrared reflection–absorption spectroscopy (IRRAS). The changes in topography and the established continuity of the layers were revealed by scanning electron microscopy (SEM), stylus profilometry (SP) and atomic force microscopy (AFM). The changes in hydrophilicity after each modification and immersion step are further examined by contact angle goniometry.

## Results and Discussion

### Surface analysis of activated titanium surfaces

The surface concentration of elements present on pristine, activated and flat titanium surfaces, as determined by XPS, is summarized in Table 1. Considerable amounts of aluminum and silicon were observed on the pristine surfaces, most likely from the polishing pastes used by the producer. In order to produce a consistent and reproducible titanium oxide surface layer, four different chemical treatments were tested in both alkaline (using alkaline piranha or 0.5 M NaOH) and acidic conditions (using mixtures of H_2_SO_4_/HCl or H_2_SO_4_/H_2_O_2_). The chemical treatments were followed by 5 min oxygen plasma treatments. The tested activation procedures significantly decreased the concentration of inorganic contaminants and caused a beneficial increase in the surface concentration of titanium and oxygen. The surfaces were free of inorganic contaminants when alkaline piranha (NH_4_OH:H_2_O_2_:H_2_O) was used for the cleaning and activation process. However, despite the rigorous chemical and oxygen plasma treatments, as well as the precautions taken during the sample preparation, it was not possible to completely avoid hydrocarbon contamination. We presume that the hydrocarbon contamination takes place mainly during the transfer of the freshly oxidized titanium samples from the plasma reactor to the desiccator. Irrespective of the surface treatment, the high resolution C 1s spectra centered at 285.0 eV lacked the expected titanium carbide contribution at 281.6 eV.

**Table 1:** Influence of the surface treatments on the surface concentration of elements present on pristine, activated and flat titanium surfaces, as determined by XPS. The ratio between the surface oxides and hydroxides determined from the analysis of the high resolution O 1s spectra is also reported.

Treatment	Ti	O	C	Al	Si	MtOH/Oxide
	(atom %)					

Pristine	7.9	50.3	28.8	7.3	5.7	0.04
NH_4_OH:H_2_O_2_:H_2_O	18.0	59.0	23.0	–	–	0.2
NaOH	17.0	58.4	21.5	3.1	–	0.1
HCl/H_2_SO_4_	17.8	58.6	19.3	4.4	–	0.1
H_2_SO_4_/H_2_O_2_	18.0	55.5	24.6	–	1.9	0.2
Flat surface	19.5	56.7	23.8	–	–	0.2

**Figure 1:**
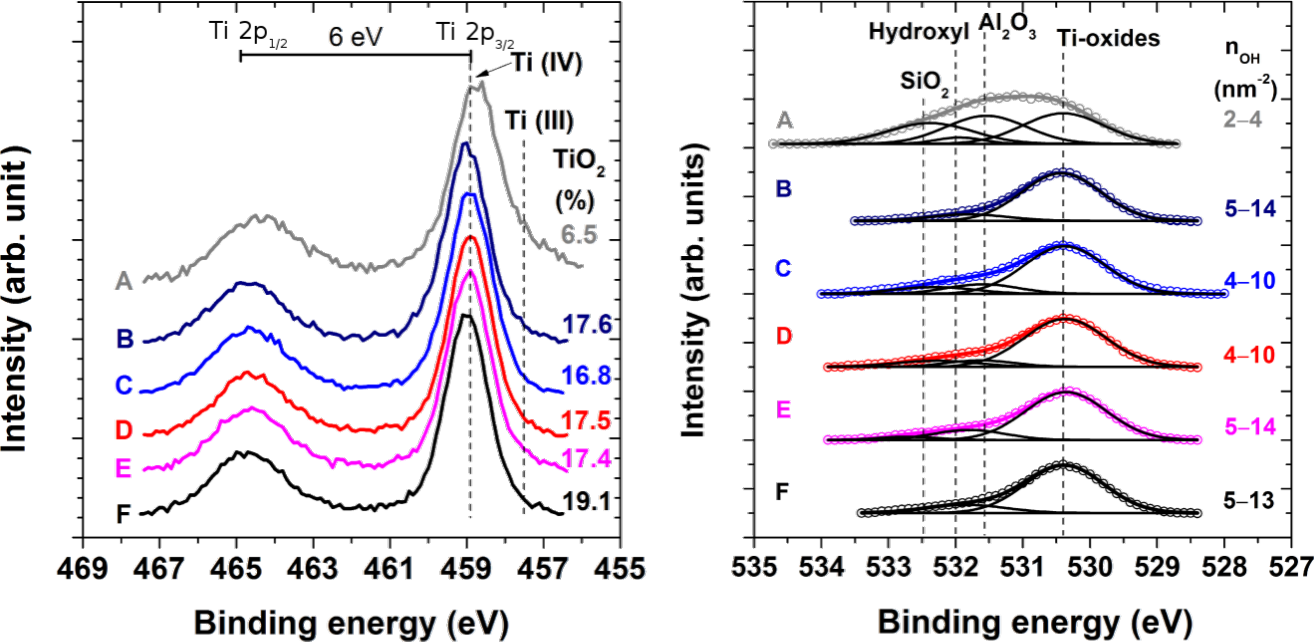
High resolution Ti 2p (left) and O 1s (right) XPS spectra of pristine titanium surfaces (A) and surfaces treated using alkaline piranha (B), 0.5 M NaOH (C), a HCl/H_2_SO_4_ mixture (D) and piranha solution (H_2_SO_4_/H_2_O_2_) (E). The spectrum of flat titanium surfaces deposited on silicon substrates (F) is given for comparison. The dominant contribution (more than 96.7%) within the Ti 2p_3/2_ envelope appears at 458.9 ± 0.1 eV and is identified as TiO_2._ The measured O 1s spectra (points) was fitted (solid, colored lines) by resolving the individual surface oxide contributions (black lines) centered at 530.3 ± 0.1, 531.5 ± 0.1 and 533.0 ± 0.3 eV arising from titanium oxides (TiO_2_ and Ti_2_O_3_), Al_2_O_3_ and SiO_2_, respectively. The hydroxy groups on the surface gave rise to the peak at 531.8 ± 0.2 eV. The figure also reports the surface density of hydroxy groups (n_OH_).

Furthermore, the surfaces were free of metallic titanium (peak at 454.1 eV). Similar to the observations on rough [19] and ultra-flat titanium surfaces performed by template striping [43], our high resolution titanium 2p spectra in the region of 450–468 eV showed the characteristic Ti 2p spin-split doublet structure, with a separation of approximately 6 eV between the Ti 2p_1/2_ and Ti 2p_3/2_ peaks (Figure 1). The binding energies of the contributions within the Ti 2p_3/2_ envelope were found at 458.9 ± 0.1 and 457.4 ± 0.1 eV and were assigned to TiO_2_ and Ti_2_O_3_, respectively. The activation treatments increased the TiO_2_ concentration from 80% for the pristine surfaces to more than 97% for the activated titanium surfaces.

Figure 1 also reports the high resolution oxygen 1s XPS spectra of the studied titanium surfaces. The O 1s envelope could be resolved into surface oxide contributions at 530.3 ± 0.1 eV, 531.5 ± 0.1 eV and 533.0 ± 0.3 eV arising from titanium oxides (TiO_2_ and Ti_2_O_3_), Al_2_O_3_ and SiO_2_, respectively. The presence of hydroxy groups on the surface was evidenced by the presence of the peak at 531.8 ± 0.2 eV. The performed activation treatments increased the contribution of the hydroxy from the observed 2% for the pristine surfaces to more than 6% for the activated titanium surfaces (Supporting Information File 1, Table S1). This was evidenced by the change in the ratio between surface hydroxy and oxide from 0.04 for the untreated titanium surfaces to values in the range 0.1–0.2 for the activated ones. The careful analysis of the obtained high resolution O 1s spectra enabled the estimation of the concentration of hydroxy groups on the titanium surfaces according the method proposed by McCafferty and Wightman [3]. The performed activation treatments increased the surface density of the hydroxy groups from the initial 2–4 hydroxy groups per nm^2^ on the pristine titanium surfaces to 4–14 hydroxy groups per nm^2^. These values are consistent with the range of values of 5–15 hydroxy groups per nm^2^ reported for titanium foils [3] and titanium dioxide powders [44]. The observed concomitant increase in concentration of surface hydroxy groups and decrease in the presence of surface contaminants inevitably leads to higher reactivity of the treated surfaces [4,44]. In addition to an increase in the number of surface sites available for binding, SEM (Supporting Information File 1, Figure S1) and stylus profilometry (Supporting Information File 1, Table S2) analysis showed an increased microscale texture for all treated surfaces (alkaline piranha, 0.5 M NaOH, and piranha (H_2_SO_4_/H_2_O_2_)) except for those treated with a H_2_SO_4_/HCl solution. Microscale texturing similar to that reported here has been obtained by treatments such as machining [45–46], anodic oxidation [45–46] and chemical oxidation using piranha [12]. The increase in the surface roughness and the creation of a specific microscale texture due to oxidative treatments as observed in our study have been shown to enhance the rate of bone formation [12,45–46].

The decreased organic contamination and increased surface density of hydroxy groups on the activated surfaces is further evidenced by the higher hydrophilicity of the treated surfaces (Supporting Information File 1, Table S2). The activation treatments decrease the measured advancing water contact angles from about 50° for the pristine titanium surfaces to values lower than 30°. Almost completely wettable surfaces were obtained when alkaline piranha was used as the surface activation treatment.

It is worth mentioning that the chemical activation using alkaline piranha simultaneously led to augmentation of the surface composition, surface reactivity, topography and hydrophilicity. Therefore, this chemical activation treatment is a potentially valuable step in the treatment of titanium surfaces and possible implants based on this material. Importantly, the evaporation-deposited, flat, titanium reference samples (*R*_RMS_ < 1.0 nm) have the same surface composition and surface density of hydroxy groups (Table 1 and Figure 1) as the activated pure titanium surfaces. Therefore, it is reasonable to consider the flat surfaces as a representative reference surface of the activated pristine titanium for the verification of the surface modifications based on thin anchor layers and on the alginate monolayer. The absence of surface irregularities on these mirror-like substrates enables techniques such as SE, IRRAS and AFM to be used for the characterization of sub- and mono-molecular, organic overlayers.

### Anchor layer deposition

The reproducibility in terms of continuity, uniformity, reactivity and adhesion properties of the anchor layers is a prerequisite for the creation of grafted adlayers with defined properties [34]. The attachment of the anchor layers of three FDA approved, organic compounds (neridronate, APTES and dopamine) were performed on oxygen plasma-activated, flat titanium substrates. As previously observed by XPS, the exposure of titanium and titanium oxide surfaces to air resulted in a thin adherent layer of organic contaminants. The presence of such an organic contaminant layer influences the optical dispersion function of the titanium films. These ill-defined optical parameters of the titanium substrates decrease the precision of the ellipsometric data analysis during subsequent surface modifications. A practical way to circumvent this problem is to perform the SE measurements in different solvents (ethanol, isobutanol, toluene), a method referred as the multiple-environment method. Due to the refractive index matching between the solvents and the adsorbed organic contaminants, and the possible dissolution of the contaminants, the multiple-environment method revealed the intrinsic optical dispersion function of the flat titanium surfaces. The measured data of a neat titanium layer in different solvents was simultaneously fitted with the parameters of a Drude–Lorentz and two Lorentz oscillator functions as discussed in Supporting Information File 1, Figure S2.

The SE analysis showed formation of a 0.9 ± 0.3 nm thick monolayer of neridronate. The concomitant processes of the self-assembly of dopamine and its intermediates (dopamine-quinone, 5,6-dihydroxyindole, etc.), of SI polymerization and of adsorption of the resulting polymer molecules resulted in a 15.2 ± 0.5 nm thick confluent PDA layer. Despite the precautions taken during the APTES deposition for the formation of the SAM [21] (freshly distilled reagents, dry titanium substrates and elevated temperature during the capping reaction), a 12.4 ± 1.7 nm thick APTES siloxane polymer multilayer was formed [21]. The presence of the anchor layers increased the water contact angles of completely wettable flat titanium surfaces (oxygen plasma treated) to 40 ± 1°, 72 ± 1° and 60 ± 5° for the substrates containing neridronate, APTES and PDA, respectively. Here, the lower hydrophilicity is caused by the presence of organic molecules with increased hydrophobicity in comparison to the neat titanium surface.

**Figure 2:**
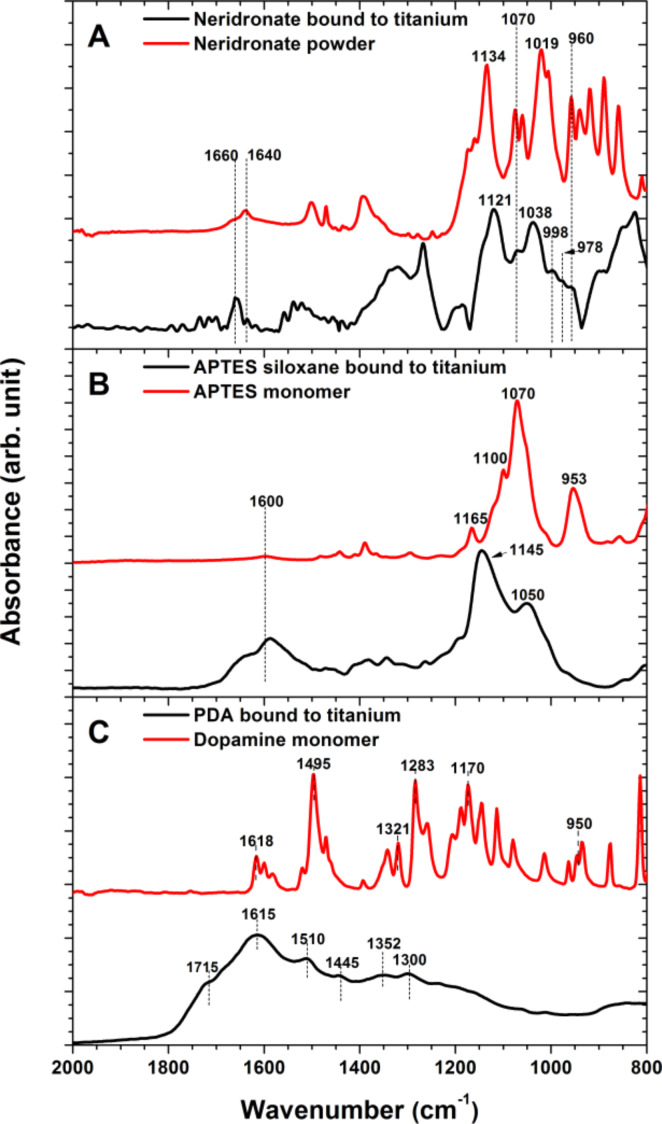
FTIR spectra of starting neridronate (A), APTES (B) and dopamine (C) organic moieties in their native state (red) and corresponding immobilized films on activated flat titanium surfaces (black). The spectra of the immobilized films were taken in IRRAS mode against backgrounds of bare titanium.

The surface immobilization of these organic moieties and their covalent structure was further verified by independent IRRAS and XPS measurements. The IR spectra of the resulting anchor layers showed the characteristic in-plane bending modes, δ_in-plane_(NH), of free and H-bonded amino groups in the region 1600–1650 cm^−1^. The covalent immobilization of the BP molecules is verified by the significant changes in the IRRAS spectra (Figure 2A) of the original sodium neridronate powder and the neridronate self-assembled monolayer on the titanium surface. Similarly to the observations on different free phosphonic acids in aqueous solution [47–48] and phosphonic acids adsorbed on bentonite [49], the 2000–800 cm^−1^ region in our IR spectrum of sodium neridronate powder was characterized by different stretching (ν) and bending (δ) modes of P=O, P–O(H) and P–O– units of sodium phosphonate and free phosphonic acid. The initially observed spectrum of the sodium neridronate powder significantly changed upon tethering of the molecules to the titanium surface. The neridronate monolayer immobilized to the titanium surface gives rise to the asymmetric and symmetric ν_as_ P–OTi of R(PO_3_), ν_s_ P–OTi of R(PO_3_), ν_s_ P–O, ν_s_ P–O of R(PO_3_^2−^), ν_as_ P–OH of R(HPO_3_^2−^) modes at 1121, 1038, 996, 978 and 960 cm^−1^, respectively. The observed absence and/or reduced intensity of the strong bands of ν_as_ (P–O) and ν_s_ (P–O) of P(OH)_2_ in the region below 960 cm^−1^ indicates that the neridronate molecules are covalently bound to the titanium surface by forming not only monodentate, but also bidentate complexes.

The IR spectrum of the liquid APTES monomer is characterized by the ν(Si–O–C) stretching modes at 1165, 1100, 1070 and 953 cm^−1^ (Figure 2B). The formation of the 12.4 ± 1.7 nm thick adherent polymer layer of APTES siloxane was further evidenced by the IRRAS measurements. The dominant vibrations of the APTES films were observed at 1145 and 1050 cm^−1^. These contributions were assigned to the ν(Si–O–Si) stretching modes. As initially described by Kurth and Bein [21], the presence of these vibrations verify not only the immobilization of the APTES molecules to the surface, but also proves the presence of the siloxane polymer network. The network is formed by SI polymerization from non-hydrolyzed ethoxy groups (which can give rise to silanol groups) and free silanols that did not undergo a dehydration condensation reaction with surface hydroxy groups. Both possible mechanisms are accompanied by adsorption and further condensation between the surface reactive species and APTES siloxane aggregates formed in the reactive solution. The presence of ethoxy groups, resulting in an incomplete cross-linking of the APTES siloxane polymer network, can be seen by the significant broadening of the dominate IR contributions toward the initially observed main peaks of APTES "monomer". This may be a source of the hydrolytical layer instability. In a water environment, the primary amines present in the network can intra- or inter- molecularly coordinate to a silicon center and catalyze the hydrolysis reaction.

The spectrum of a solid dopamine monomer (Figure 2C) is characterized by skeletal vibration modes of aromatic double bonds (1650–1400 cm^−1^), stretching ν(C–O) modes of the catechol moieties at 1283 cm^−1^, in-plane bending δ_in-plane_(C–H) at 1170 cm^−1^ and stretching modes ν(C–C–N) of the aminoethyl chain at 935 cm^−1^ [50]. The oxidative polymerization of dopamine and the surface attachment of different monomer units (dopamine-quinone, 5,6-dihydroxyindole, etc.) caused evident changes in the IR spectra (Figure 2C) and resulted in a confluent layer of PDA [32–34,51]. Similar to previous studies on PDA modified materials [32–34,52], the spectrum of PDA immobilized onto titanium substrates is characterized by poorly resolved bands of many overlapping vibration modes of the different monomer units. The most prominent contributions at 1615, 1510, 1445 cm^−1^ originate from the C=C vibrations of the different monomer units, whereas the shoulder at 1715 cm^−1^ indicates the presence of quinone groups. The shift in frequency, the broadening of the initial contributions, as well as the appearance of new bands with respect to the IR spectra of dopamine, proves not only the polymer nature of the resulting films, but also their complex highly conjugated covalent structure.

**Figure 3:**
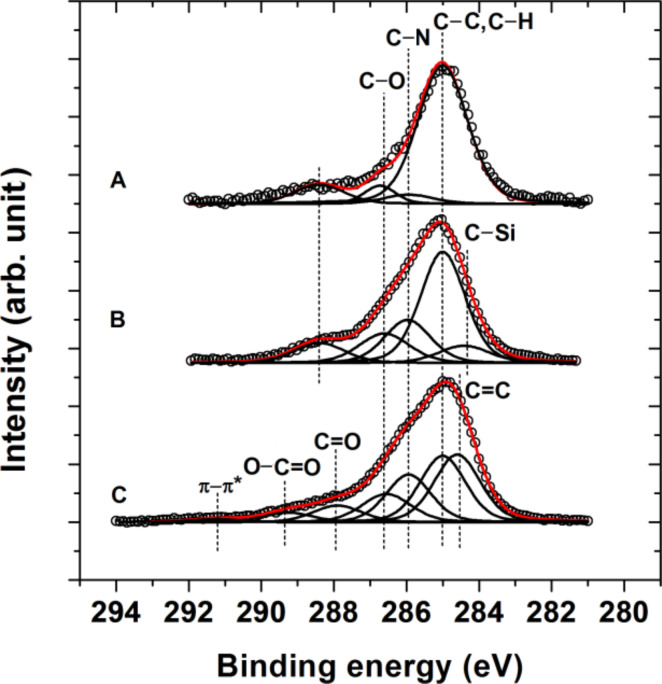
High resolution C 1s XPS spectra of neridronate (A), APTES siloxane (B) and PDA (C) films on the surfaces of activated flat titanium substrates (black). The unfilled circles represent the measured data, while the red lines represent the fitted data. The individual contributions to the fitted data of different functional groups present in the films are represented with black lines.

The complementary XPS measurements further verified the successful formation of surface adherent films and their covalent structure. The determined elemental compositions of the neridronate, APTES siloxane and PDA anchor layers is reported in Table 2. The covalent tethering of the organic moieties caused an increase in the contributions of carbon and was associated with the significant decrease in the surface concentration of titanium. In the case of the thick polymer anchor films of APTES and PDA, the contributions arising from the titanium substrate were negligible. Importantly, the XPS spectra verified the presence of nitrogen on the surface of different anchor layers. Moreover, the obtained relative ratios N/P = 0.57 for the neridronate and N/Si = 0.65 for APTES siloxane are reasonably close to the expected values of 0.5 and 1, respectively. Figure 3 reports the high resolution carbon 1s XPS spectra for the neridronate, APTES siloxane and PDA adherent films immobilized on the flat titanium surfaces. The C 1s envelope of the anchor layers could be resolved into contributions centered at 285 ± 0.1 eV arising from sp^3^ carbon (C–C and C–H functionalities), at 285.9 ± 0.1 eV arising from the C–N species of amines and at 286.6 ± 0.2 eV arising from the C–O contribution of hydroxy groups present in neridronate, the non-hydrolyzed ethoxy groups of APTES and catechols of poly(dopamine). The spectrum of APTES has an additional contribution at 284.3 ± 0.2 eV from the C–Si functionality. The PDA shows contributions at 284.5 ± 0.1, 288.0 ± 0.2, 289.3 ± 0.3 and 291.2 ± 0.2 eV arising from the carbon species of sp^2^ carbon (C=C functionality), the C=O functionality of the quinons, the carboxylic carbon functionality (O–C=O groups) and the π–π* transition (shake-up), respectively [53–54]. A rather unexpected peak at 288.4 ± 0.1 eV was observed in the high resolution C 1s spectra of neridronate and APTES siloxane. Although with a large uncertainty, Acres et al. have tentatively assigned this peak to the C–C=O functionality [55]. A similar contribution was observed for micro-plasma polymerized APTES layers [56] and was attributed to amide contribution. Since our immobilization protocols lack harsh plasma deposition treatments, we tentatively attribute this functionality to carbamate-like structures, which form due to the scrubbing effect of amines on CO_2_ from air [57].

**Table 2:** Elemental compositions of anchor layers (neridronate, APTES siloxane and PDA) and ALG layers tethered to these surfaces, as determined by XPS.

Modification	Ti	O	C	N	P	Si
	(atom %)					

Neat, flat titanium surface	19.5	56.7	23.8	–	–	–
Neridronate	13.8	55.6	25.9	1.7	3.0	–
APTES siloxane	–	27.1	54.6	7.2	–	11.1
PDA	0.4	22.1	70.7	6.8	–	–
ALG/neridronate	1.8	22.2	70.1	5.8	–	–
ALG/APTES siloxane	0.6	27.9	60.5	6.3	–	4.7
ALG/PDA	–	30.7	61.9	7.4	–	–

**Figure 4:**
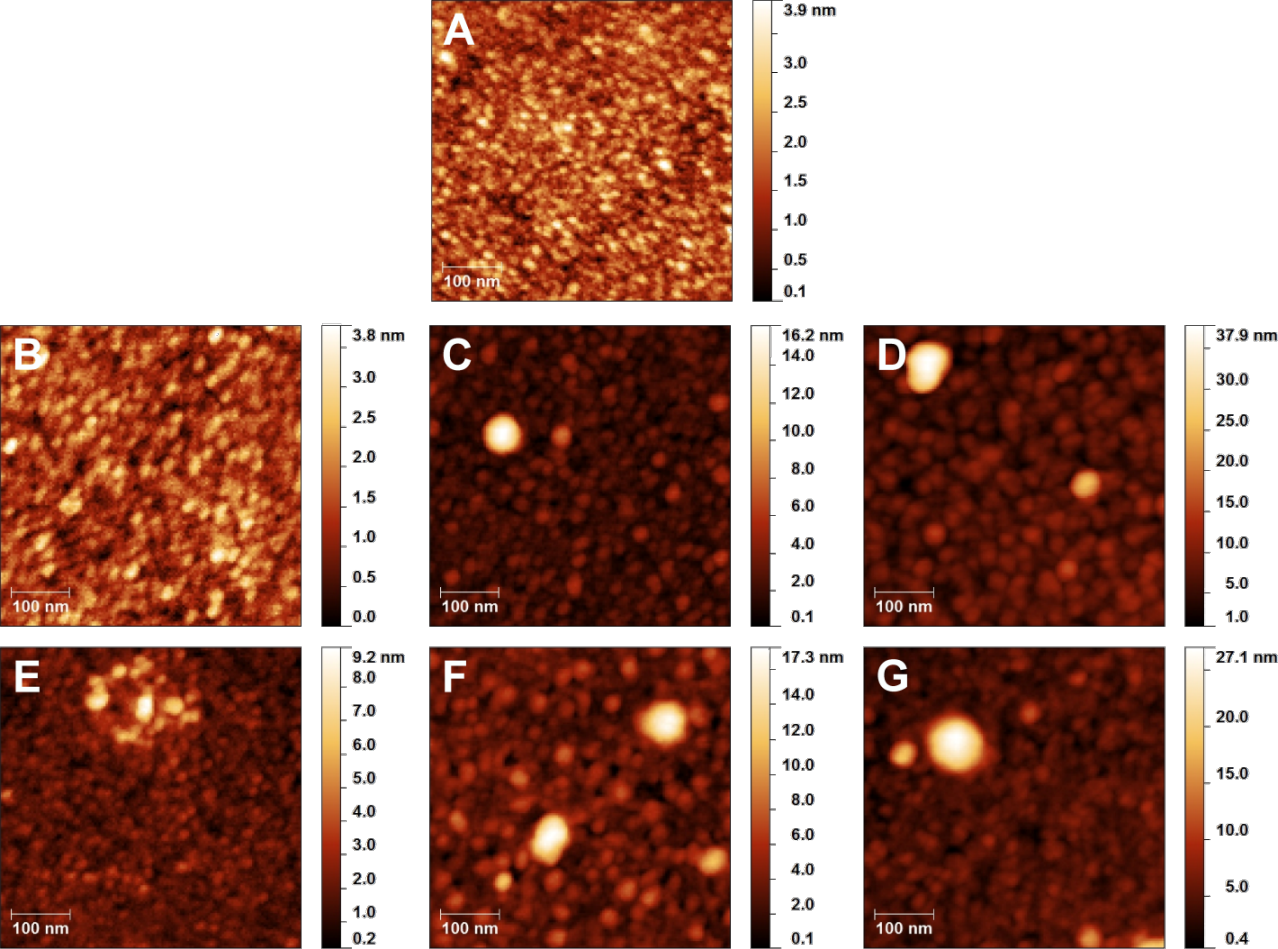
AFM images of the neat, flat titanium surface (*R*_RMS_ = 0.5 ± 0.3 nm) (A), and confluent anchor layers of neridronate (*R*_RMS_ = 0.5 ± 0.2 nm) (B), APTES (*R*_RMS_ = 1.1 ± 0.2 nm) (C) and PDA (*R*_RMS_ = 3.6 ± 1.2 nm) (D). The figure also reports AFM images of ALG layers grafted onto neridronate (*R*_RMS_ = 0.7 ± 0.3 nm) (E), APTES (*R*_RMS_ = 1.8 ± 0.2 nm) (F) and PDA (*R*_RMS_ = 2.9 ± 1.0 nm) (G) anchor layers. The AFM measurements on the ALG surfaces were performed on predominantly flat regions with *R*_RMS_ values similar to those of the initial anchor layers.

The topography and homogeneity of the resulting anchor layers on titanium substrates was monitored via AFM. The corresponding images are presented in Figure 4. The AFM data clearly evidence the functionalization of the flat titanium surfaces with confluent anchor layers free of pinholes. The self-assembly of neridronate molecules resulted in a fine-grained topography similar to the activated titanium surface (Figure 4A and Figure 4B). The processes of self-assembly, SI polymerization and adsorption of aggregates from the reactive solution during adsorption and formation of the APTES siloxane and PDA layers led to surfaces with an increased roughness of 1.1 ± 0.2 nm and 3.6 ± 1.2 nm, respectively (Figure 4C and Figure 4D). The immobilized polymer surfaces exhibited a more pronounced grain structure with nanoparticles having average diameter of 12 nm for the APTES siloxane and of 38 nm for PDA anchor layers. While such a pronounced surface topography is considered to be an inherent characteristic of the PDA films [34,53], the presence of such surface objects on APTES layers is rarely discussed. However, even in the case when a APTES SAM was achieved [58], the presence of surface adherent objects resembling aggregates having average diameter of up 30 nm was inevitable. We presume that the observed APTES siloxane aggregates are formed in the reactive solution and further adsorbed and even grafted onto the surface.

### Covalent binding of alginate

The tethering of alginate (ALG) chains by reaction of the carboxyl groups to the amine-functionalized titanium surfaces was performed by following standard EDC/NHS protocols. The binding reaction resulted in the formation of 5.0 ± 1.9 nm thick alginate films, irrespective of the anchor layer. The grafting density, calculated from the ellipsometric thickness and the molecular weight of ALG (1.5 × 10^6^ g∙mol^−1^), was 1.3–3.0 × 10^11^ chains/cm^2^. The presence of the polysaccharide chains was obvious from the significant increase in the surface hydrophilicity. The immobilization of hydrophilic alginate chains resulted in a decrease in the contact angles from the values determined for the anchor layers to 13 ± 3°, 19 ± 3° and 13 ± 1° for the alginate films bound to neridronate, APTES and PDA, respectively.

**Figure 5:**
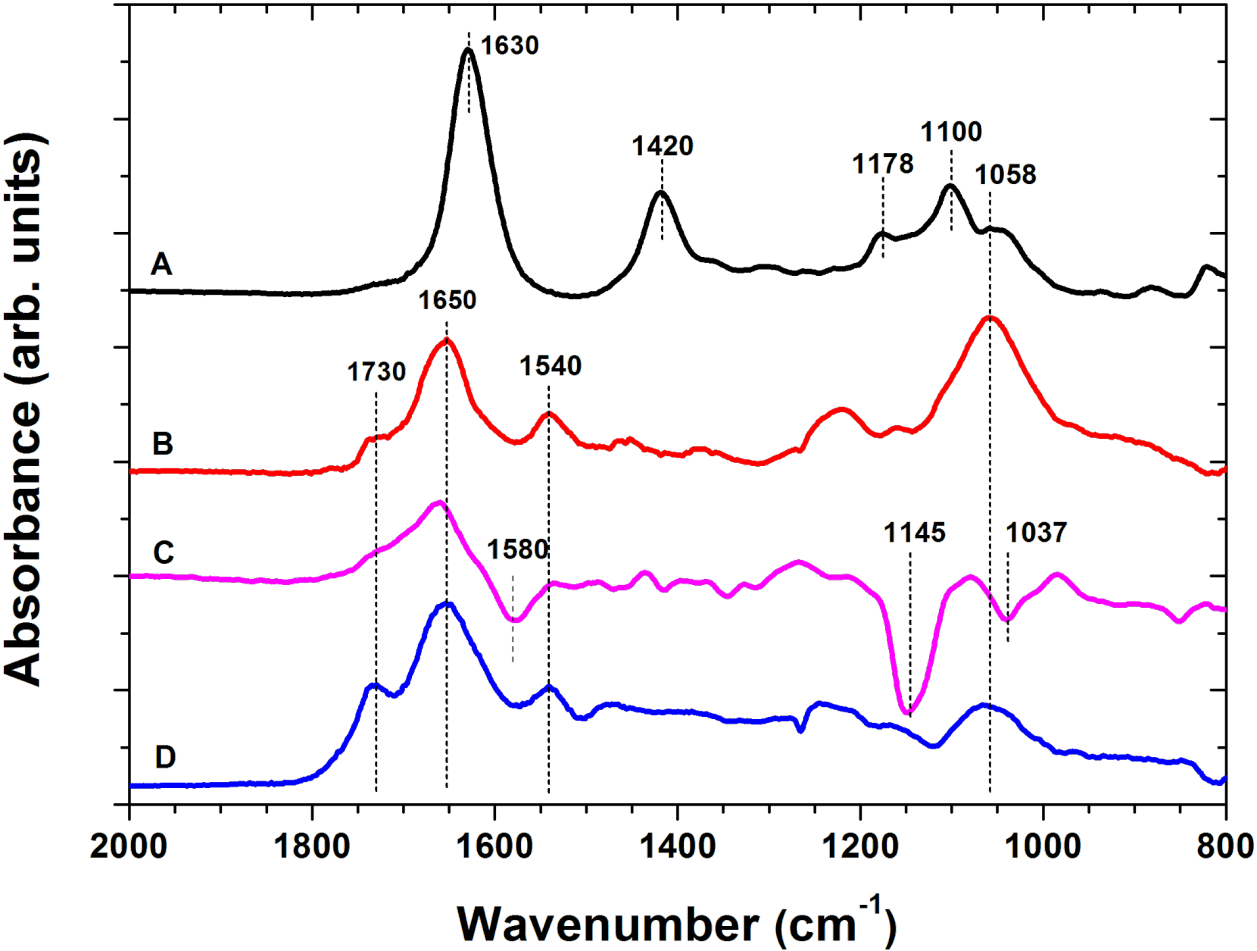
Differential IRRAS spectra of free alginate adsorbed onto a flat titanium surface (A) and covalently bound alginate molecules to the amines of the neridronate (B), APTES (C) and PDA (D) anchor layers. The IRRAS spectra of covalently bound alginate films was characterized by the presence of the carbonyl band (1730 cm^−1^), amide I (1650 cm^−1^), amide II band (1540 cm^−1^) and ν(C–O) stretching modes of the pyranosyl ring, β-(1-4)-glycosidic bonds and hydroxy groups of the polysaccharide (1200–1000 cm^−1^). The spectra were referenced to corresponding background spectra of bare titanium and titanium bearing different anchor layers.

The covalent tethering of the polysaccharide chains and the formation of amide bonds between the activated carboxyl groups of ALG and the amines present in the anchor layer were probed by IRRAS measurements. A representative IR spectrum of free alginate deposited on the flat titanium surface is presented in Figure 5. The spectrum is dominated by the symmetric ν_sym_ (C=O) and asymmetric ν_asym_ (C=O) modes of charged carboxyl groups at 1630 and 1420 cm^−1^, respectively, in addition to the stretching ν(C–O) modes of the pyranosyl ring, β-(1-4)-glycosidic bonds and hydroxy groups of the polysaccharide in the 1200–1000 cm^−1^ region. The established covalent bonds between the carboxyl groups of the alginate chains and the amines present on the surface are evidenced by the appearance of the highly specific amide I and amide II bands at 1650 and 1540 cm^−1^, respectively (Figure 5). Additionally, the differential spectra of ALG bound to neridronate and PDA show the carbonyl band (1730 cm^−1^) and the bands characteristic for the polysaccharide moieties (1200–1000 cm^−1^). In the same region, the differential spectrum of ALG bound to the siloxane anchor layer is characterized by a valley at 1580 cm^−1^ arising from decreased in-plane bending δ_in-plane_(NH) of amine contributions and at 1145 and 1037 cm^-1^ from decreased ν(Si–O–Si) stretching contributions. The appearance of these bands is associated with the amine-catalyzed hydrolysis of the siloxane bonds of the polymer network [22] during the 24 h immersion in MES buffer.

**Figure 6:**
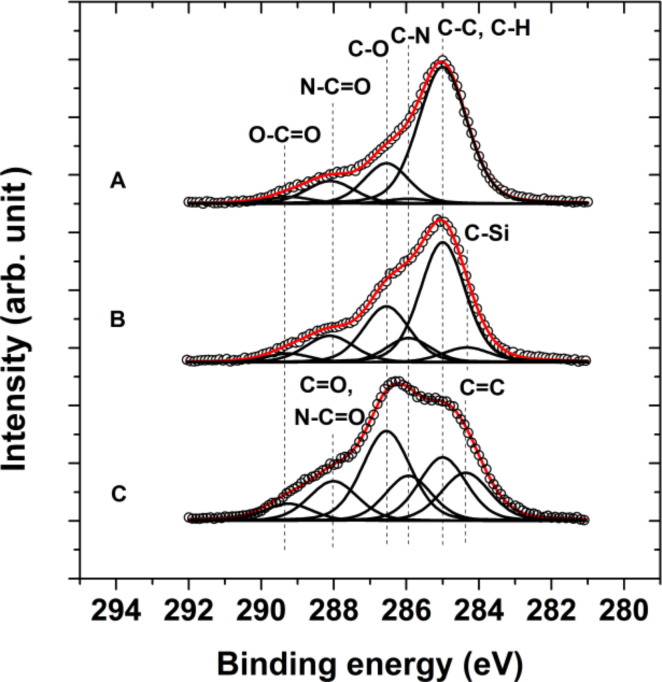
High resolution C 1s XPS spectra of alginate coatings on neridronate (A), APTES siloxane (B) and PDA (C) anchoring layers. The unfilled circles represent the measured data, while the red lines represent the fitted data. The individual contributions to the fitted data of different functional groups present in the films are represented with black lines.

The IRRAS results of the immobilization of ALG to the anchor layers are further supported by XPS measurements. The formation of the 5 nm thick polysaccharide layer on the surfaces with different anchors serves to further decrease the contributions from the titanium substrate. This corresponds to a concomitant increase in the presence of the elements from the organic moieties (Table 2). Compared to the high resolution C 1s spectra of the anchor layers (Figure 3), the spectra of the bound alginate films (Figure 6) show increased contributions at 286.5 ± 0.1 eV arising from the C–O moiety of the pyranosyl ring, β-(1-4)-glycosidic bonds and hydroxy groups of the polysaccharide in addition to a peak at 289.2 ± 0.1 eV arising from the O–C=O functionality of carboxylic groups. Importantly, the presence of the peak at 288.1 ± 0.2 eV verifies the formation of amide bonds (N–C=O) between the activated carboxyl groups of ALG and the amines present in the anchor layers. Thus, the XPS studies strongly prove the covalent immobilization of the alginate films.

The tethering of ALG resulted in surfaces with a predominantly flat topography (Figure 4E–G) similar to those observed for the corresponding anchor layers (Figure 4B–D). The ALG films on neridronate, APTES siloxane and PDA were characterized by *R*_RMS_ values of 0.7 ± 0.3 nm, 1.8 ± 0.2 nm and 2.9 ± 1.0 nm, respectively. The increase in the ellipsometric thickness of 5 nm combined with the AFM findings of the surface roughness (similar to the values characteristic for the anchor layers) indicates the formation of continuous ALG films, which merely replicate the surface underneath. However, although only occasionally observed, the AFM measurement also showed the presence of regions of surface-immobilized ALG aggregates composed of particles with an average diameter exceeding 40 nm (Supporting Information File 1, Figure S3). The observed nanoparticle aggregates may have a physisorbed fraction of loosely bound ALG chains that are a potential source of instability and defects when these surfaces are exposed to physiological conditions.

### Stability of alginate films

The stability of the anchor layers and anchored ALG films is crucial for their performance especially when biomedical and tissue engineering applications are in question. The deterioration of these surface confluent layers could affect the surface concentration of free carboxylic end groups that are essential in the envisaged applications. Furthermore, the instability of the layers that are in intimate contact with the titanium surface could result in complete delamination of the potentially surface adherent alginate gels.

**Figure 7:**
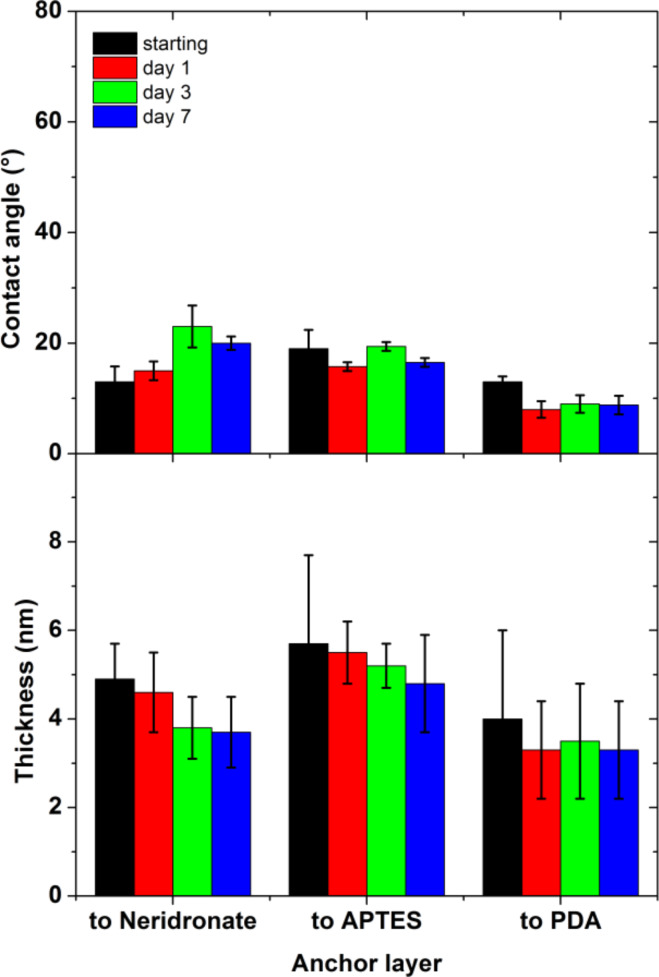
Ellipsometric thickness and water contact angle evolution of ALG bound to neridronate, APTES siloxane and PDA during the immersion in PBS (37 °C, pH = 7.4) (mean value ± SD, n = 15).

The stability tests of the neat, anchor layers and the ALG/anchor layers on flat titanium substrates were performed by immersion in PBS buffer at 37 °C for a period of 7 days. The ellipsometric thickness and water contact angles were measured on dry films after 1, 3 and 7 days of incubation (Figure 7 and Supporting Information File 1, Figure S4). The thickness of the ALG adlayers was obtained from an optical model that considered a constant thickness of the neridronate and PDA anchor layers as determined before the grafting. The adopted optical model conforms with the observed stability of neat neridronate and PDA films during the immersion in PBS (Supporting Information File 1, Figures S4 and S5). When the polysaccharide layer was bound to the APTES siloxane, the optical model considered the instability of the APTES anchor in accordance with the SE, CA (Supporting Information File 1, Figure S4) and IRRAS findings (Supporting Information File 1, Figure S5). This methodology enabled monitoring of the stability of the ALG adlayer in the two-layer stack.

**Figure 8:**
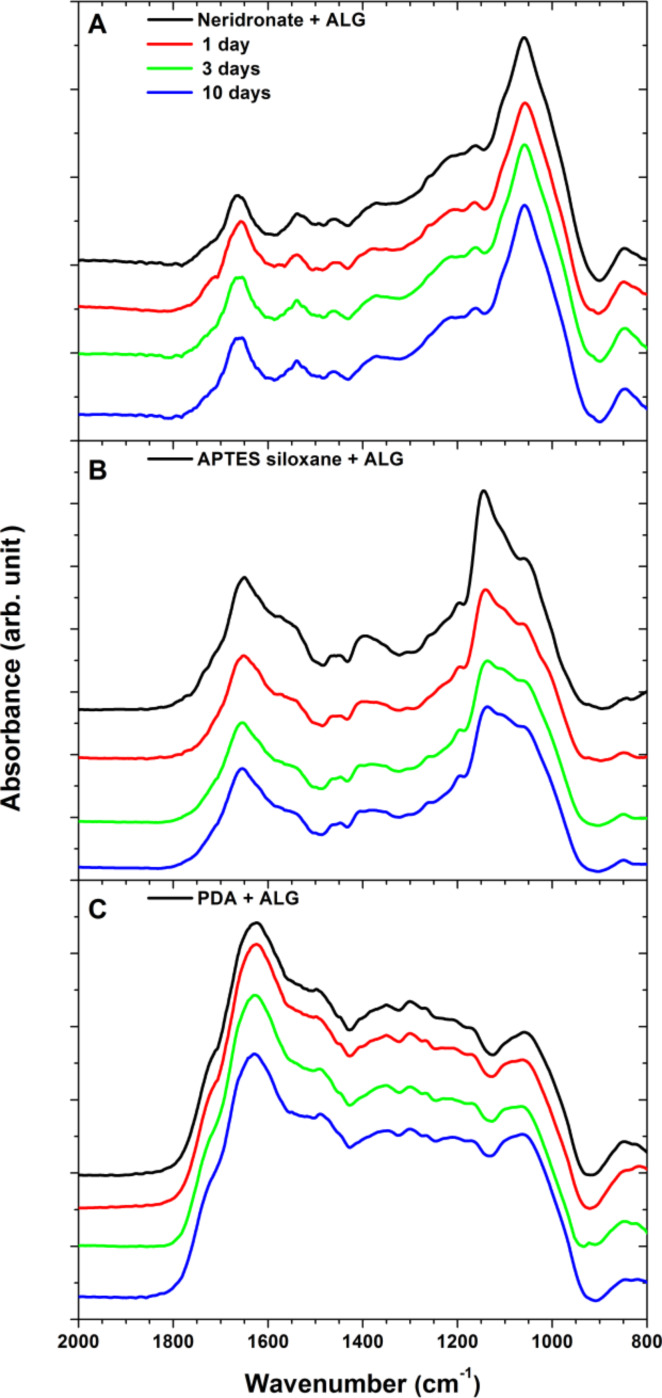
Evolution of IRRAS spectra of ALG bound to neridronate (A), to APTES siloxane (B) and to PDA (C) upon immersion in PBS at 37 °C for 7 days.

During the 7 days of incubation, only a minor decrease in the measured ellipsometric thickness was observed (Figure 7). We presume that the reduction in the ALG thickness is mainly caused by the release of a physisorbed fraction present in the surface adherent ALG aggregates. However, the water contact angles of the ALG coatings remained rather constant. This indicates that the observed, small decrease in thickness due to deterioration of the ALG films does not reveal the less-wettable anchor layers underneath (Supporting Information File 1, Figure S4). The stability of the ALG/anchor layers and the neat anchor layers was further verified by IRRAS measurements (Figure 8 and Supporting Information File 1, Figure S5). The IRRAS data enabled monitoring of the changes in the covalent structure of the whole ALG/anchor double layer and allowed for the contributions of both constituents of the stack to be separately resolved. As depicted in Figure 8, the polysaccharide films bound to neridronate and PDA anchors are rather stable without any significant changes in the position and intensity of the main vibrations of the double layer components. However, the ALG films anchored to APTES showed continuous deterioration during the 7 days of immersion. The main reduction in intensity was observed for the bands centered at 1146 and 1046 cm^−1^ arising from the ν(Si–O–Si) stretching modes of the APTES siloxane polymer network. The position of these bands corresponds to the positions of the decreased contributions in the IRRAS spectra of the deteriorated neat APTES siloxane films (Supporting Information File 1, Figure S5). This implies that the main components released during the immersion are of siloxane nature. At the same time, the spectral contributions of ALG were affected to a minor extent, most likely due to the several grafting points through which the polysaccharide chains are bound to the surface. However, the previously discussed hydrolytic instability of the APTES siloxane anchor layer would eventually lead to a complete cleavage of the polysaccharide layer from the titanium surface during a long-term immersion.

Based on the stability observations, the alginate monolayers bound to the neridronate or PDA anchor layers can be potentially used for the immobilization of a thin alginate hydrogel carrier of bioactive compounds (such as calcium phosphates or other biologically active molecules) formed by ionic cross-linking [40]. The proposed architecture is envisaged to enhance adhesion, proliferation, differentiation of osteoblasts, and thus ultimately, to achieve a better integration of the titanium implant into the bone tissue.

## Conclusion

In the present contribution, we demonstrated the successful covalent attachment of ALG chains to neridronate, APTES and PDA anchor layers immobilized on activated titanium surfaces. The formation of the ALG and anchor layer films was investigated utilizing SE, AFM and contact angle goniometry. The IRRAS analysis further evidenced the established amide bonds between the carboxyl groups of ALG and amine groups of the anchor layers. The immobilization of the organic moieties, as well as the changes in the surface composition of pristine titanium surfaces after different surface activation treatments, was probed by XPS measurements. The changes in the surface morphology and roughness parameters during the activation of titanium surfaces were monitored by SEM and SP analysis. The 5 nm thick ALG layers anchored to neridronate and PDA were stable during immersion under physiological-like conditions for 7 days. The hydrolysis of the anchoring APTES siloxane network led to a higher deterioration tendency of the ALG/APTES double layer. The presented surface modification strategy of titanium can be an effective path for the formation of ALG-based hydrogel coatings enriched with bioactive compounds for bone tissue engineering applications.

## Experimental

### Materials

Dopamine hydrochloride (98.5%) was purchased from Sigma and 3-triethoxysilylpropan-1-amine from Aldrich. 3-(Ethyliminomethyleneamino)-*N*,*N*-dimethylpropan-1-amine (EDC), 1-hydroxy-2,5-pyrrolidinedione (NHS) and 2-(morpholin-4-yl)ethanesulfonic acid (MES) were obtained from Fluka. Sodium alginate salt (ALG) derived from brown algae was purchased from Sigma. The molecular weight of ALG was determined by size exclusion chromatography (SEC) on a gradient Knauer system with diode array detection (DAD) and an Alltech 3300 evaporative light scattering detection (ELSD) system. The SEC measurement was performed on a PolySept GFC-P linear column using an isocratic system of 0.03 M ammonium acetate buffer in acetonitrile/water (20/80 v/v). The determined average molecular weight of ALG was 1.5 × 10^6^ g·mol^−1^ (PDI = 2.45) with the column calibration carried out using PEO standards. The ALG peak had a unimodal distribution without the presence of low molecular weight degradation products.

All organic solvents (petroleum ether, methanol, ethanol, isobuthanol and toluene) were of analytical grade (Lach-Ner, Czech Republic) and used as received. Ultrapure water was obtained with a Millipore Milli-Q system.

### Substrate preparation

Clean, single-side-polished silicon wafers (CZ, orientation <100>, B-doped, resistivity 5–20 Ω∙cm) with a ≈50 nm SiO_2_ thermal overlayer (Siegert Consulting e.K., Germany) were used as substrates for the preparation of ultraflat titanium surfaces. Flat, titanium reference surfaces (50 nm thickness) were obtained by evaporation deposition (rate = 0.35 Å·s^−1^, pressure = 6.66 × 10^−6^ Pa) using a COV AP SQC-310C deposition device (Angstrom Engineering, Canada). The coated substrates were cut into 1.2 cm × 1.2 cm pieces, sonicated in methanol, and deionized in water for 15 min, followed by oxygen plasma oxidization (25 W, Plasma Cleaner/Sterilizer, Harrick, USA) for 5 min directly before the anchor layer immobilization.

**Scheme 1:**
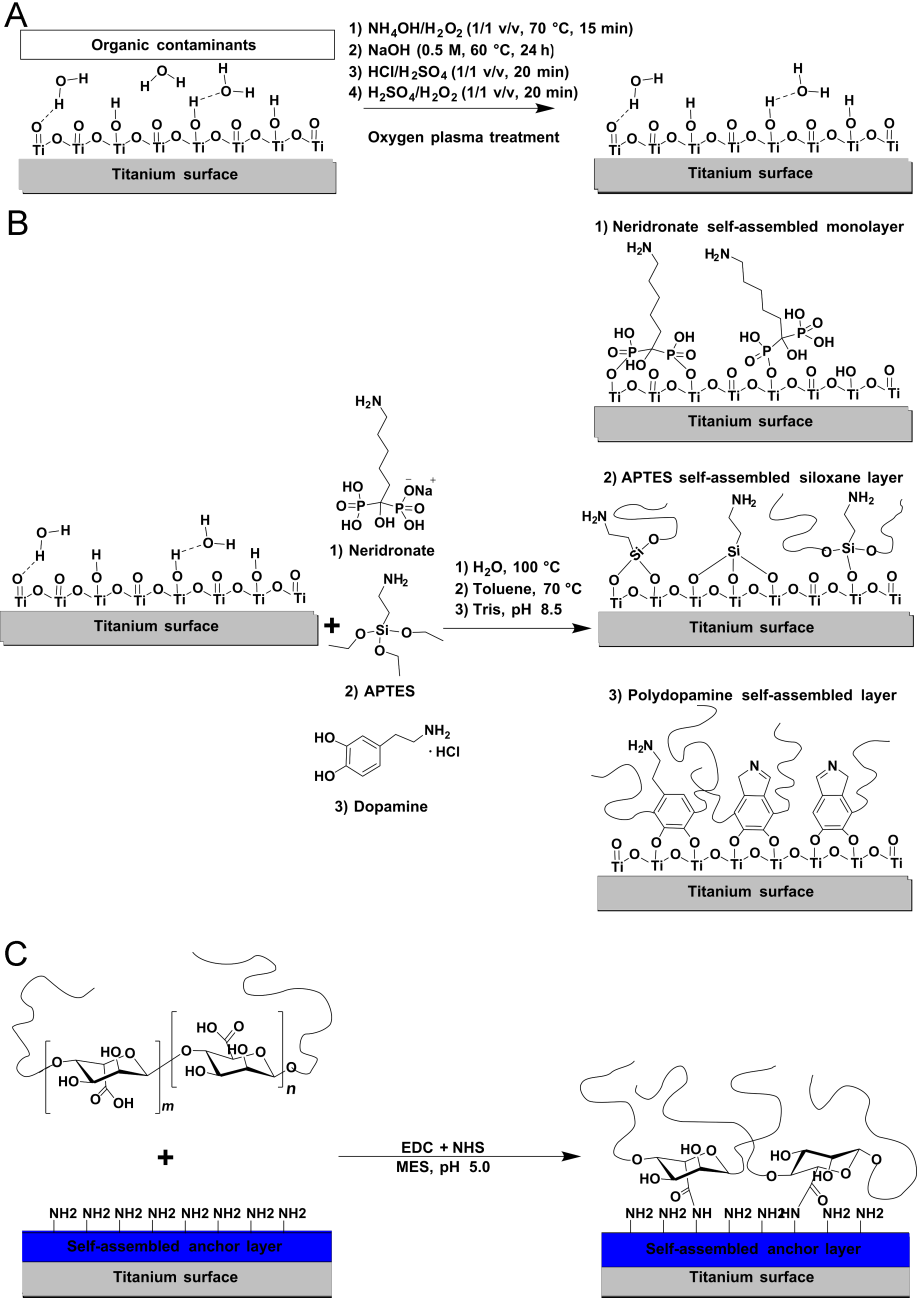
Performed surface treatments and subsequent reactions for the activation and modification of titanium surfaces. (A) Cleaning and activation procedures for the removal of inorganic and organic contaminants from the titanium surface and to increase the number of free hydroxy groups. (B) Immobilization of neridronate, APTES siloxane and poly(dopamine) anchor layers through surface specific reactions between the phosphonate, silane and catechol groups of corresponding compounds and hydroxy groups on the surface. (C) Covalent binding of ALG chains to amino groups present in the anchor layers by the EDC/NHS coupling reaction.

Commercially available, paste-polished, pure titanium surfaces (Beznoska, Czech Republic) were used to probe the presence of inorganic and organic surface contaminants and to determine the surface concentration of introduced hydroxy groups. After the initial sonication in petrolether, methanol and deionized water for 15 min, the following surface cleaning and activation procedures were investigated: alkaline piranha treatment (mixture of 25% NH_3_, 30% H_2_O_2_ and water at 1:1:5 v/v/v, at 70 °C for 15 min), immersion in 0.5 M NaOH (60 °C for 24 h), immersion in a mixture of concentrated HCl and H_2_SO_4_ (1:1 v/v, at room temperature for 20 min) and a piranha cleaving treatment utilizing concentrated H_2_SO_4_ and 30% H_2_O_2_ (1:1 v/v, at room temperature for 20 min). The substrates were subsequently thoroughly rinsed with ultrapure water, blow-dried using nitrogen, and exposed to an oxygen plasma (25 W) for 5 min just before the XPS analysis or the binding of the anchor layers (Scheme 1).

### Formation of anchoring layers

Neridronate monosodium salt ((6-amino-1-hydroxy-1-phosphonohexyl)-hydroxyphosphinate sodium) was prepared according previous reports [59]. The immobilization on the flat titanium surfaces proceeded from a 0.005 M neridronate solution in water at 100 °C for 48 h. Afterwards, the samples were rinsed in water 3 times for 5 min to remove the physisorbed molecules, then blow-dried and kept in vacuum until further use.

Siloxane anchor layers were prepared by exposing the activated titanium substrates to 0.1% v/v APTES solutions in dry toluene at 70 °C. After 12 h of exposure, the samples were sonicated in dry toluene for 15 min to remove the physisorbed siloxane particles, then blow-dried and kept in vacuum until further use.

A poly(dopamine) coating was deposited from a 2 mg·mL^−1^ solution prepared by dissolution of dopamine hydrochloride in an air-saturated 10 mM Tris hydrochloride (pH 8.5) buffer. After 3 h of polymerization, the PDA-coated surfaces were rinsed with water, sonicated in water for 15 min and blow-dried in a stream of nitrogen.

Stability tests were performed on alginate-containing and neat-anchor layers deposited on flat titanium substrates, incubated in PBS (pH 7.4, containing 0.02 wt % sodium azide) at 37 °C for 7 days. After the immersion period, the substrates were rinsed with copious amounts of water and blow-dried in a stream of nitrogen.

### Tethering of alginate onto titanium layers containing anchor layers

The tethering of the alginate chains to the amine-functionalized flat titanium substrates was performed employing a modified EDC/NHS protocol based on the work of Rowley et al. [60]. Alginate was dissolved in mixture of 0.1 M MES and 0.15 M NaCl, at pH 5, at a concentration of 1 wt %. Next, EDC was added and the solution was stirred for 15 min. Afterwards, NHS was added and the solution was stirred for an additional 15 min until the production of bubbles diminished. The molar ratio between reactants was uronic units/EDC/NHS (1:20:20). The titanium substrates bearing the neridronate, APTES siloxane and PDA anchors were placed in 12-well cultivation plates and 0.8 mL of the reaction solution was deposited on the surface and allowed to react for 24 h. Subsequently the alginate-grafted substrates were rinsed with water and blow-dried in a stream of nitrogen.

### Methods

Spectroscopic ellipsometry (SE): Ex situ and in situ ellipsometric data were acquired using a spectroscopic imaging, auto-nulling ellipsometer (EP^3^-SE, Nanofilm Technologies, Germany) equipped with a liquid cell (*V*_internal_ = 0.7 mL) in 4-zone mode in the wavelength range of 398.9–811.0 nm (source: Xe arc lamp, wavelength step: 10 nm) at an angle of incidence of 60°. The cell windows (strain-free, optical BK-7 glass from Qioptiq, Germany) exhibited only small birefringence and dichroism causing errors in the ellipsometric angles Δ and Ψ smaller than 0.3° and 0.1°, respectively. These errors were corrected following the method of Azzam and Bashara [61]. To increase the measurement precision and exclude errors from the variations of layer thickness throughout the substrate area, a 10× objective and position-calibrated sample stage were utilized to perform repeated ex situ and in situ measurements over the same sample area (1 × 2 mm). The obtained data were analyzed with multilayer models using the EP^4^-SE analysis software (Accurion GmbH, Germany).

The thickness and refractive index of the resulting organic layers were obtained from simultaneous fitting of the obtained ellipsometric data using the Cauchy dispersion function (*n* = A_n_ + B_n_/λ, *k* = 0 with A_n_ = 1.412 ± 0.010, B_n_ = 5900 ± 80 nm^2^ for neridronate and A_n_ = 1.413 ± 0.009, B_n_ = 6270 ± 70 nm^2^ for APTES and A_n_ = 1.4714 ± 0.008, B_n_ = 13200 ± 1000 nm^2^ for the ALG layers). The optical dispersion functions of PDA, silicon dioxide and silicon were taken from previous reports [34,62]. The optical dispersion functions of ethanol, isobutanol, toluene and titanium dioxide were taken from the EP^4^-SE database.

Contact angle measurement*:* The wettability of the organic surfaces on flat, titanium reference surfaces was examined by a static sessile water drop method using a DataPhysics OCA 20 contact angle system. Each sample was characterized using four 3 μL drops of material. The data were evaluated using the Young–Laplace method.

The wettability of the commercially available, rough titanium substrates upon different treatments was estimated by measuring the advancing and receding water contact angles utilizing the dynamic Wilhelmy plate method. The measurements were performed on a Kruss K12 (Germany) tensiometer.

Infrared reflection–absorption spectroscopy (IRRAS): The infrared spectra of neridronate, APTES and dopamine moieties were recorded using a Perkin Elmer, Paragon 1000PC, FTIR spectrometer, equipped with a MCT detector and a single reflection, monolithic diamond, Golden Gate ATR accessory (Specac, England). The spectra of the dry organic films formed on titanium surfaces were recorded using a Bruker IFS 55 FTIR spectrometer (Bruker Optics, Germany) equipped with a MCT detector. The measurements were performed at a grazing angle (80°, p-polarization) using the reflection spectroscopy accessory. The measurement chamber was continuously purged with dry air. The acquisition time was approximately 20 min at a resolution of 2 cm^−1^. The spectra are reported as −log(*R*/*R*_0_), where *R* is the reflectance of the sample and *R*_0_ is the reflectance of bare titanium reference surfaces.

Atomic force microscopy (AFM): AFM characterization was performed on a Dimension ICON (Bruker, USA) system in peak force tapping mode in air using silicon probes (TAP150A, Bruker, USA) with a typical force constant of 5 N∙m^−1^. The images were taken using a scan rate in the range of 0.5−1.2 Hz and a peak force set point of 0.02−0.2 V.

Surface profilometry: Macroscopic surface roughness and waviness measurements were performed using a Tencor P-10 (Texas, USA) surface profiler with 1 mm long scans at a speed of 20 μm∙s^−1^ and a sampling rate of 200 Hz using a maximum stylus force of 0.02 N.

Scanning electron microscopy (SEM): The SEM analysis was performed on a Quanta 200 FEG (FEI, Czech Republic) microscope. All micrographs presented are secondary electron images taken under high vacuum using an accelerating voltage of 30 kV.

X-ray photoelectron spectroscopy (XPS): The core-level photoelectron spectra were recorded using an angle-resolved photoelectron spectrometer, ADES 400 (VG Scientific, UK), operating at a base pressure of 1.33 × 10^−7^ Pa. The system was equipped with an X-ray excitation source and a rotatable hemispherical electron energy analyzer. The photoelectron spectra were recorded using Mg Kα radiation with a pass energy of 100 eV or 20 eV (high-energy resolution). The incidence angle was 70° with respect to the sample surface normal and the emission angle along the surface normal. The atomic concentrations of carbon, oxygen and nitrogen were determined from the C 1s, O 1s, and N 1s photoelectron peak areas after a Shirley inelastic background subtraction. Assuming a simple model of a semi-infinite solid of homogeneous composition, the peak areas were corrected for the photoelectric cross-sections [63], the inelastic mean free paths of the electrons in question [64], and the transmission function of the spectrometer [65]. The experimental uncertainties in the quantitative analysis of XPS were assessed in separate experiments with several standard materials and were estimated to be below 7%. This value encompasses the overall uncertainties of the method that are typically introduced by the background subtraction.

## Supporting Information

File 1 Additional Experimental Information.
