# Supplementary material for: Self-assembled anchor layers/polysaccharide coatings on titanium surfaces: a study of functionalization and stability
Source: Beilstein J Nanotechnol. 2015 Mar 2;6:617–31. doi: 10.3762/bjnano.6.63 (PMC4362089; doi:10.3762/bjnano.6.63)
Supplement: File 1 — Additional Experimental Information. [file Beilstein_J_Nanotechnol-06-617-s001.pdf]

# Supporting information

# for

# Self-assembled anchor layers/polysaccharide coatings on titanium surfaces: a study of functionalization and stability

Ognen Pop-Georgievski[1,*,§], Dana Kubies[1,§], Josef Zemek[2], Neda Neykova[2,3], Roman Demianchuk[1], Eliška Mázl Chánová[1], Miroslav Šlouf[1], Milan Houska[1] and František Rypáček[1]

Address: [1]Institute of Macromolecular Chemistry, Academy of Sciences of the Czech Republic, Heyrovsky sq. 2, 16206 Prague 6, Czech Republic, [2]Institute of Physics, Academy of Sciences of the Czech Republic, Cukrovarnicka 10, 16253 Prague 6, Czech Republic and [3]Czech Technical University in Prague, Faculty of Nuclear Sciences and Physical Engineering, Trojanova 13, 12000 Prague 2, Czech Republic

Email: Ognen Pop-Georgievski - georgievski@imc.cas.cz

* Corresponding author

[§] These authors contributed equally

# Additional Experimental Information

**Table S1:** Influence of the surface treatments on the surface concentration of titanium oxides, $Al_2O_3$, $SiO_2$ and hydroxyl groups as determined by peak fitting of the O 1s XPS spectra reported in Figure 2. The contributions are reported with respect to the total surface composition (Table 1).

| Treatment | Total O1s | Ti oxides | $Al_2O_3$ | $SiO_2$ | Hydroxyls |
|---|---|---|---|---|---|
| | (atom %) | | | | |
| Pristine | 50.3 | 18.2 | 16.7 | 13.1 | 2.3 |
| $NH_4OH:H_2O_2:H_2O$ | 59.0 | 50.0 | – | – | 9.0 |
| NaOH | 58.4 | 43.9 | 8.1 | – | 6.4 |
| $HCl/H_2SO_4$ | 58.6 | 47.0 | 5.4 | – | 6.2 |
| $H_2SO_4/H_2O_2$ | 55.5 | 44.7 | – | 2.2 | 8.7 |
| Flat surface | 56.7 | 48.9 | – | – | 7.9 |

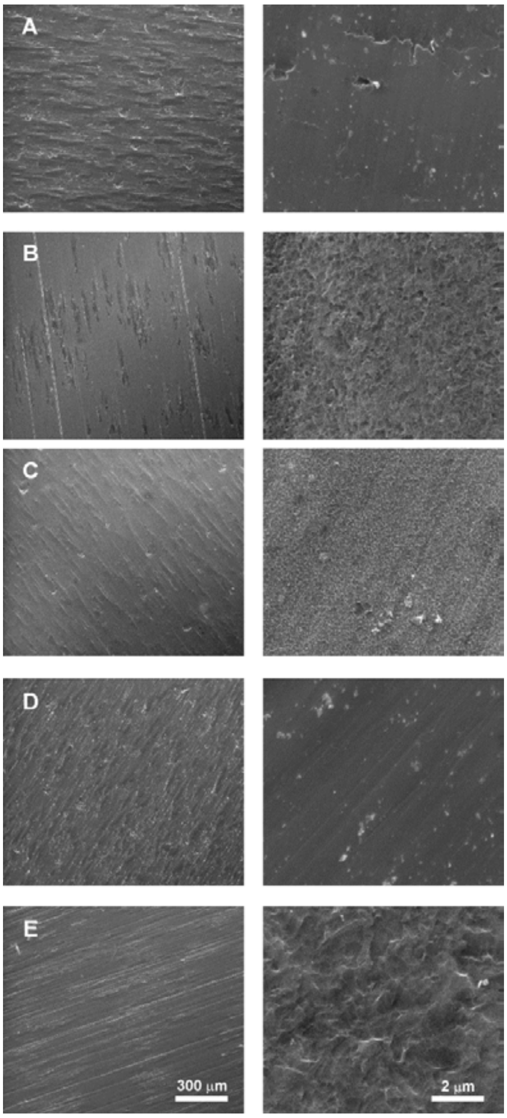


**Figure S1:** Scanning electron microscopy of pristine titanium surfaces (A) and surfaces treated using alkaline piranha (B), 0.5 M NaOH (C), mixture of concentrated acid (HCl and $H_2SO_4$) (D) and piranha (concentrated $H_2SO_4$ and 30% $H_2O_2$) (E). The scale bar for the left micrographs is 300 μm the right is 2 μm. All treated samples show uniform topography. While the treatment using a mixture of concentrated acids resulted in surfaces with unchanged surface morphology, the other activation treatments gave surfaces with increased microtexture. Such a microstructure has been reported to have an inherent ability to increase the rate of bone formation.

**Table S2:** Changes in root mean squared roughness ($R_{RMS}$) and root mean squared waviness ($W_{RMS}$) due to the surface treatments as determined by stylus profilometry. The resulting changes in surface wettability, that is, advancing ($\theta_A$) and receding ($\theta_R$) contact angle between the samples and water, were tested by the Wilhelmy plate method.

| Treatment | $R_{RMS}$ (nm) | $W_{RMS}$ (nm) | $\theta_A$ (°) | $\theta_R$ (°) |
|---|---|---|---|---|
| Pristine | 40 ± 10 | 330 ± 150 | 52 | 12 |
| $NH_4OH:H_2O_2:H_2O$ | 140 ± 20 | 650 ± 180 | 3 | –[a] |
| NaOH | 50 ± 30 | 500 ± 230 | 27 | 13 |
| $HCl/H_2SO_4$ | 60 ± 20 | 610 ± 220 | 14 | 12 |
| $H_2SO_4/H_2O_2$ | 190 ± 60 | 780 ± 200 | 15 | –[a] |

[a]Missing values signify complete wetting during measurement of the receding water contact angle

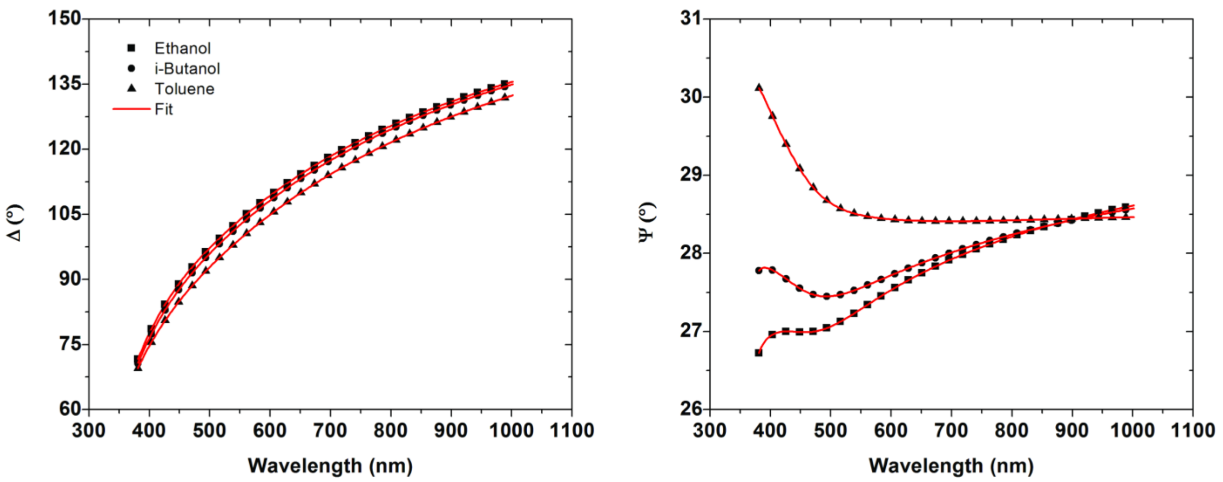


**Figure S2:** Multiple environment ***in situ*** spectroscopic ellipsometry. Δ and Ψ ellipsometric spectra of neat titanium surfaces were measured in ethanol (■), isobutanol (●) and toluene (▲). The red lines represent the fitted data. The measured data of a neat titanium layer in different solvents was simultaneously fitted with the parameters of a Drude–Lorentz model (amplitude: 24.268 $eV^2$, broadening: 1.648 eV) and two Lorentz oscillator functions ($E_1$ = 2.808 eV with amplitude: = 5.191 $eV^2$, broadening: 1.585 eV; $E_2$ = 2.998 eV with amplitude: *28.077* $eV^2$, broadening: 1.585 eV).

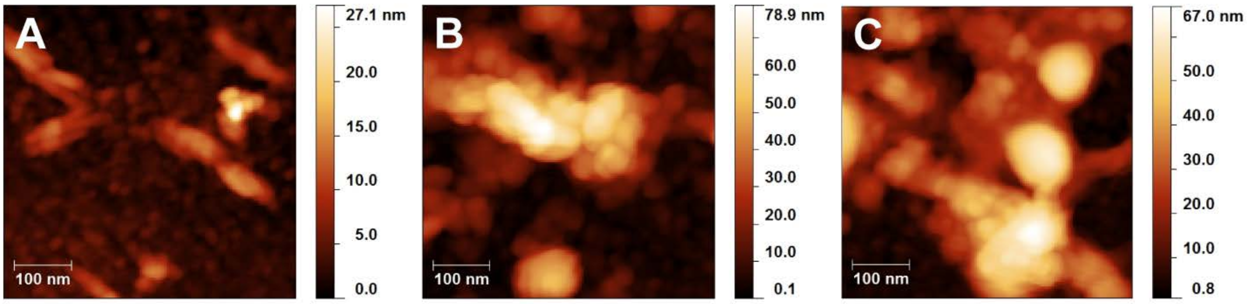


**Figure S3:** AFM images of ALG films immobilized on neridronate (A), APTES (B) siloxane and PDA (C) anchor layers. The AFM measurements were performed on regions with pronounced topography arising from surface immobilized aggregates of alginate. The determined roughness of these films was in the range of 5–15 nm.

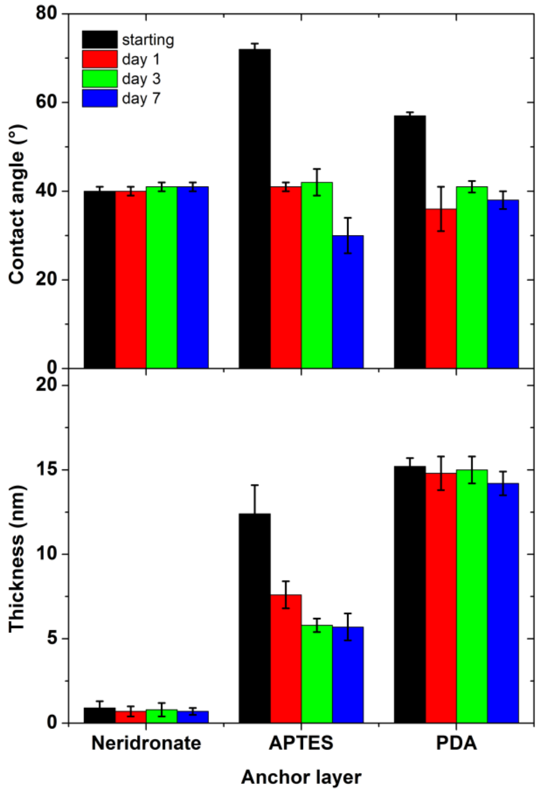


**Figure S4:** Ellipsometric thickness and water contact angle evolution of neridronate, APTES siloxane and PDA during the immersion in PBS (37 °C, pH = 7.4) (mean value ± SD, n = 15). The pronounced decrease in thickness and water CA during the immersion indicate strong deterioration of the APTES siloxane layers. The changes in the water CA of PDA are most probably a result of structural reorganization.

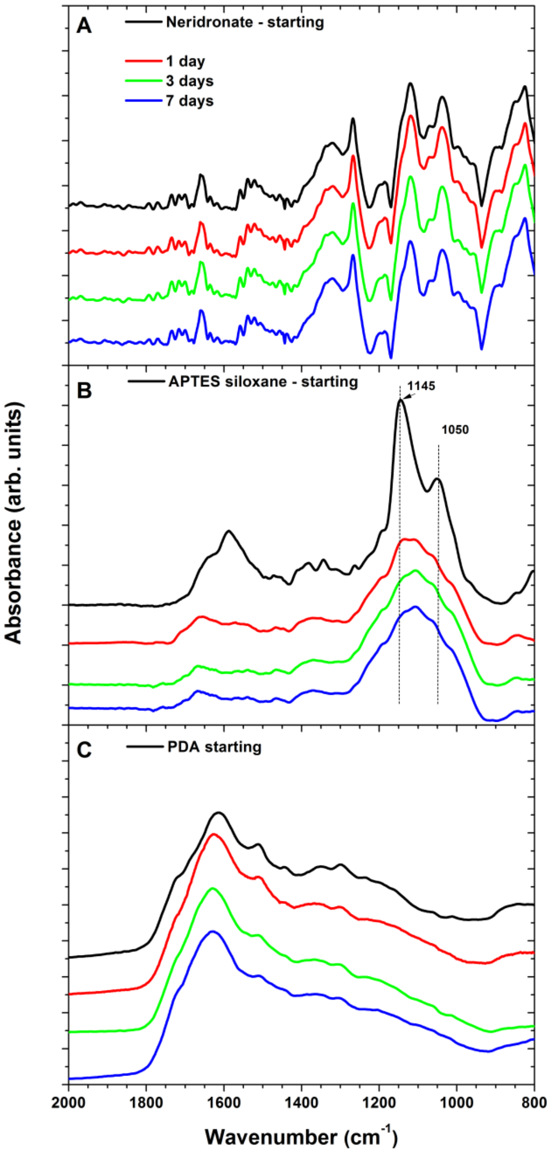


**Figure S5:** Evolution of IRRAS spectra of neridronate (A), APTES siloxane (B) and PDA (C) upon immersion in PBS at 37 °C for 7 days.
